# Urinary Activin A is a novel biomarker reflecting renal inflammation and tubular damage in ANCA-associated vasculitis

**DOI:** 10.1371/journal.pone.0223703

**Published:** 2019-10-15

**Authors:** Yoshinori Takei, Shunsuke Takahashi, Masao Nakasatomi, Toru Sakairi, Hidekazu Ikeuchi, Yoriaki Kaneko, Keiju Hiromura, Yoshihisa Nojima, Akito Maeshima

**Affiliations:** 1 Department of Nephrology and Rheumatology, Gunma University Graduate School of Medicine, Maebashi, Japan; 2 Department of Nephrology and Rheumatology, Japanese Red Cross Hospital, Maebashi, Japan; University of Louisville, UNITED STATES

## Abstract

Activin A, a member of the transforming growth factor-beta superfamily, is a critical modulator of inflammation and plays a key role in controlling the cytokine cascade that drives the inflammatory response. However, the role of activin A in inflammatory kidney diseases remains unknown. To address this issue, we examined here whether activin A can be detected in the kidney and/or urine from patients with antineutrophil cytoplasmic antibody (ANCA) -associated vasculitis (AAV). Fifty-one patients who had been diagnosed with AAV and were treated in our department between November 2011 to March 2018 were included in this study. Forty-one patients had renal complications (renal AAV). Serum and urinary activin A levels were measured by enzyme-linked immunosorbent assay. Correlation of urinary activin A concentration with clinical parameters was analyzed. Urinary activin A was undetectable in healthy volunteers. In contrast, urinary activin A concentration was significantly increased in patients with renal AAV but not in those with non-renal AAV. Urinary activin A concentration decreased rapidly after immunosuppressive treatment. There was a significant correlation of urinary activin A level with urinary protein, L-FABP, and NAG. Histologic evaluation revealed that urinary activin A levels were significantly higher in patients with cellular crescentic glomeruli than in those lacking this damage. In situ hybridization demonstrated that the mRNA encoding the activin A βA subunit was undetectable in normal kidneys but accumulated in the proximal tubules and crescentic glomeruli of the kidneys of patients with renal AAV. Immunostaining showed that activin A protein also was present in the proximal tubules, crescentic glomeruli, and macrophages infiltrating into the interstitium in the kidneys of patients with renal AAV. These data suggested that urinary activin A concentration reflects renal inflammation and tubular damage in AAV and may be a useful biomarker for monitoring renal AAV.

## Introduction

Activin is a member of the transforming growth factor-beta superfamily, a group of proteins that regulate the growth and differentiation of cells in various organs [1]. Activin A has been shown to act as a negative regulator of branching morphogenesis during kidney organogenesis [2, 3]. This protein also inhibits ureteric bud branching in embryonic kidney culture [4–7]. Blockade of activin action induces renal tubulogenesis in an in vitro 3D tubulogenesis model [8]. Consistent with these data, the number of glomeruli is increased in the kidneys of transgenic mice overexpressing a truncated activin type II receptor [9]. In adult kidneys, activin A inhibits the regeneration of renal tubules after ischemic injury [10–12]. Activin A acts as a potent inducer of renal fibrosis [13, 14] and also is involved in the development of glomerulonephritis [15], lupus nephritis [16], and acute kidney injury [17].

Recent studies have shown that activin A is an important regulator of inflammation [18] [19] and plays a key role in controlling the cytokine cascade during the development of various inflammatory diseases [20], including apparent roles in inflammatory arthropathies [21, 22], inflammatory bowel disease in mice [23], lung inflammation in cystic fibrosis patients [24], airway remodeling in asthmatic mice [25], and in fibroblast cell culture [26].” However, there exist (to our knowledge) no reports regarding the role of activin A in inflammatory kidney diseases.

In the present study, we measured urinary activin A concentrations in patients with AAV. We found that activin A levels were significantly increased in the urine of patients with renal AAV but in not in those with non-renal AAV; these activin A levels decreased rapidly after immunosuppressive treatment. Both the activin A-encoding mRNA and the protein were accumulated in the proximal tubules and crescentic glomeruli in the kidneys of patients with AAV. These findings suggest that measurement of urinary activin A may be useful for the assessment of renal inflammation and tubular damage in patients with renal AAV.

## Methods

### Patients

This study enrolled fifty-one patients with antineutrophil cytoplasmic antibody (ANCA) -associated vasculitis (AAV) who were treated in Gunma University Hospital from November 2011 to March 2018. This study included patients with first-onset AAV (n = 40), with relapsed AAV (n = 5), and with AAV in remission (n = 6). Urine samples from healthy volunteers also were used. For immunohistochemical analysis, renal biopsy samples from patients with renal AAV were used. Normal kidney specimens from patients who underwent nephrectomy because of renal cancer were used as controls. This study was approved by the ethical committee on human research of Gunma University Graduate School of Medicine (Approval numbers 855 and 15–104). Written informed consent was obtained from all patients.

### Serum and urine collection

Blood for serum and urine were collected from individual patients before treatment or at 1, 3, 6, and 12 months after the initiation of immunosuppressive treatment. Blood was centrifuged at 3,000 rpm for 10 min; the resulting serum supernatant was transferred to a fresh tube and stored at -20°C until the day of analysis. Urine samples were centrifuged at 10,000 rpm for 5 min; the resulting supernatant was transferred to a fresh tube and also stored at -20°C until the day of analysis.

### ELISA

Serum and urinary activin A concentrations were quantified by enzyme-linked immunosorbent assay (ELISA) according to the manufacturer’s instructions (Kit No. DAC00B; R&D Systems Inc., Minneapolis, MN).

### Immunohistochemistry

Immunostaining was performed using a VECTASTAIN ABC-kit (Vector Laboratories, Burlingame, CA) as described previously [10]. Briefly, paraffin-embedded sections (4-μm thicknesses) were deparaffinized and hydrated according to standard methods, soaked in blocking serum, and incubated with primary antibody overnight at 4°C. After washing with phosphate-buffered saline (PBS), sections were incubated with peroxidase-conjugated secondary antibody followed by color development with diaminobenzidine. Primary antibodies used in this study were as follows: mouse monoclonal anti-human CD68 antibody (M081401) (DAKO, Denmark) and mouse monoclonal anti-human CD163 antibody (NCL-L-CD163) (Leica, Newcastle, UK). Quantification of CD68-positive cells or CD163-positive cells was performed by counting the number of positive cells in five randomly selected fields of kidney biopsy specimens at ×400 magnification.

Fluorescent staining was performed as follows [11]. Briefly, sections were incubated with primary antibodies overnight at 4°C. After washing with PBS, sections were incubated with fluorescently-labeled secondary antibodies (Alexa, Molecular Probes, Inc., Eugene, OR) and 4’, 6-diamidino-2’-phenylindole dihydrochloride (DAPI; to stain nuclear DNA). Fluorescent images were recorded as described previously [11]. For the immunostaining control, the primary antibody was replaced with PBS, which did not show positive staining, confirming specificity. Primary antibodies used in this study were as follows: mouse monoclonal anti-CD68 antibody (ab955) (Abcam, Cambridge, MA), rabbit anti-inhibin beta A antibody (ab97705) (Abcam, Cambridge, UK), and fluorescein *Lotus tetragonolobus* lectin (LTL) (FL-1321) (VECTOR Laboratories, Burlingame, CA).

### In situ hybridization

In situ hybridization was performed using a InHyb In Situ Hybridization Kit (BioChain Institute Inc., Newark, CA) [17]. Hybridization probes were obtained from Genostaff Co., Ltd. (Tokyo, Japan). After deparaffinization and rehydration using standard methods, sections were fixed in 4% paraformaldehyde in Diethylpyrocarbonate (DEPC)-PBS at room temperature for 20 min. After digestion with 10 μg/ml Proteinase K at 37°C for 20 min, sections were post-fixed in 4% paraformaldehyde in DEPC-PBS at room temperature for 15 min. Sections were incubated with pre-hybridization solution for 3 hr at 50°C. Hybridization was performed with sense or antisense probes (2.5 ng/μl) at 50°C for 16 hr. After hybridization, sections were washed once in 2 × saline-sodium citrate (SSC) at 45°C for 10 min, 1.5 × SSC at 45°C for 10 min, and washed twice in 0.2 × SSC at 37°C for 20 min. Sections were incubated in 1× blocking solution for 60 min at room temperature and then incubated in a 1:200 diluted solution of alkaline phosphatase (AP)-conjugated anti-digoxigenin antibody for 1 hr, before washing and detection of the label with nitroblue tetrazolium chloride and 5-bromo-4-chloro-3-indolyphosphate.

### Histological examination

Using PAS or HE-stained sections, interstitial injuries and interstitial cell infiltration were microscopically evaluated and graded on a scale of 0–3 as follows: 0, normal; 1, slight; 2, moderate; and 3, severe.

### Statistical analysis

Statistical analysis was performed using SPSS Statistics 24.0 (Chicago, IL, USA). Differences between means were compared using a two-tailed, non-paired Welch's *t* test. Correlation was analyzed with Spearman’s rank correlation test coefficients. *P* values of <0.05 were considered significant.

## Results

### Baseline characteristics of the patients with AAV

Baseline characteristics of the patients with AAV are shown in Table 1. Twenty-four patients with AAV were male and twenty-seven were female; their mean age was 67.6 ± 1.8 years (mean ± SE). Forty-three patients had microscopic polyangiitis, six had granulomatosis with polyangiitis, and two had eosinophilic granulomatosis with polyangiitis. Forty-one patients had renal complications. There was no significant difference in C-reactive protein (CRP) level between patients with renal AAV and those with non-renal AAV. Serum creatinine levels and the ratio of urinary protein to urinary creatinine (U-pro/Cr) levels were significantly higher, and estimated glomerular filtration rate (eGFR) levels were significantly lower, in patients with renal AAV compared to those with non-renal AAV.

**Table 1:** Baseline characteristics of patients with AAV.

	Total	Renal	Non-renal	P value (Renal vs Non-renal)
Number	51	41	10	
Age (years)	67.6 ± 1.8	68.6 ± 1.9	63.7 ± 5.3	0.4
Sex (M/F)	24/27	20/21	4/6	
MPA/GPA/EPGA	43/6/2	35/6/0	8/0/2	
MPO+/PR3+	46/5	36/5	10/0	
Onset	40	30	10	
Focal/Crescentic/ Mixed/Sclerotic	4/4/5/1	4/4/5/1	-	
Relapse	5	5	-	
Remission	6	6	-	
CRP (mg/dl)	6.9 ± 0.7	7.1 ± 0.8	6.2 ± 1.4	0.61
sCr (mg/dl)	2.05 ± 0.3	2.39 ± 0.35	0.63 ± 0.05	<0.001
eGFR (mL/min/1.73m^2^)	44.9 ± 4.2	34.5 ± 3.3	87.6 ± 7.5	<0.001
U-Pro/Cr (g/gCr)	1.08 ± 0.18	1.32 ± 0.21	0.16 ± 0.02	<0.001

MPO: myeloperoxidase-ANCA. PR3: proteinase3-ANCA. CRP: C-reactive protein. L-FABP: L-type fatty acid-binding protein. NAG: N-acetyl-beta-glucosaminidase. MPA: microscopic polyangiitis. GPA: granulomatosis with polyangiitis. EPGA: eosinophilic granulomatosis with polyangiitis.

### Urinary activin A levels in patients with AAV

**Fig 1:**
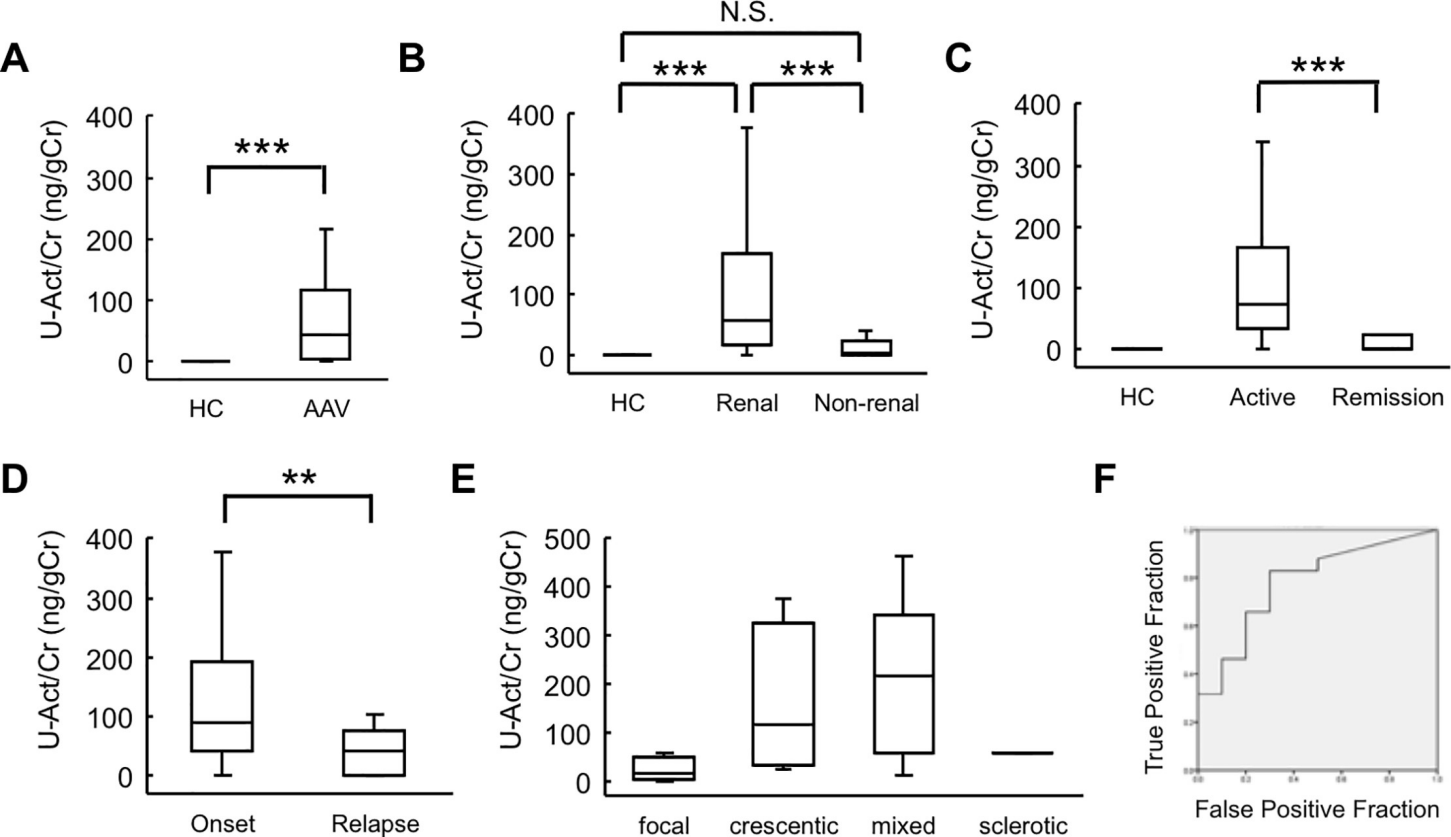
Urinary activin A levels in patients with AAV. A: Urinary activin A levels in healthy controls (HC) (n = 8) and patients with AAV (n = 51). ***p<0.001. B: Urinary activin A levels in patients with renal AAV (n = 41) and with non-renal AAV (n = 10). ***p<0.001, N.S., not significant. C: Urinary activin A levels in patients with active (onset and relapse) renal AAV (n = 35) and in patients with renal AAV in remission (n = 6). ***p<0.001. D: Urinary activin A levels in patients with first-onset renal AAV (n = 30) and in patients with relapsed AAV (n = 5). **p<0.01. E: Urinary activin A levels in patients with different histopathologic classification of glomerular crescents. F: ROC curves of urinary activin A levels in patients with renal AAV (n = 41) and with non-renal AAV (n = 10).

We first examined whether ELISA could detect activin A in the urine of patients with AAV. Activin A was undetectable in the urine of healthy control patients. In contrast, activin A was readily detected in the urine of patients with AAV (median, interquartile range [IQR], 44.6 [4.3, 114.8]) (ng/gCr); the difference was significant compared to the control patients (Fig 1A). Next, we compared urinary activin A levels between patients with renal AAV and those with non-renal AAV. Urinary activin A levels in patients with renal AAV (median [IQR], 57.6 [15.7, 167.0]) (ng/gCr) were significantly higher than those in patients with non-renal AAV (median [IQR], 2.1 [0, 24.9]) (ng/gCr) (Fig 1B). Comparison between patients with active (onset and relapse) AAV (median [IQR], 74.0 [32.1, 167.4]) (ng/gCr) and patients with AAV in remission (median [IQR], 1.3 [0, 23.0]) (ng/gCr) revealed that urinary activin A levels were significantly higher in the former group (Fig 1C). Urinary activin A levels in patients with first-onset AAV (median [IQR], 89.0 [40.8, 194.0]) (ng/gCr) were significantly higher than that in patients with relapsed AAV (median [IQR], 43.6 [0.7, 74.8]) (ng/gCr) (Fig 1D). There were no significant association between urinary activin A level and histopathologic classification of glomerular crescents (Fig 1E). The AUC of urinary activin A was 0.779 (Fig 1F); cut off: 10.7 ng/gCr; sensitivity: 82.9%; specificity: 70% and PPV, NPV, PLR, NLR: 0.918, 0.5, 2.76, 0.244, respectively.

### Changes in urinary activin A levels after treatment

**Fig 2:**
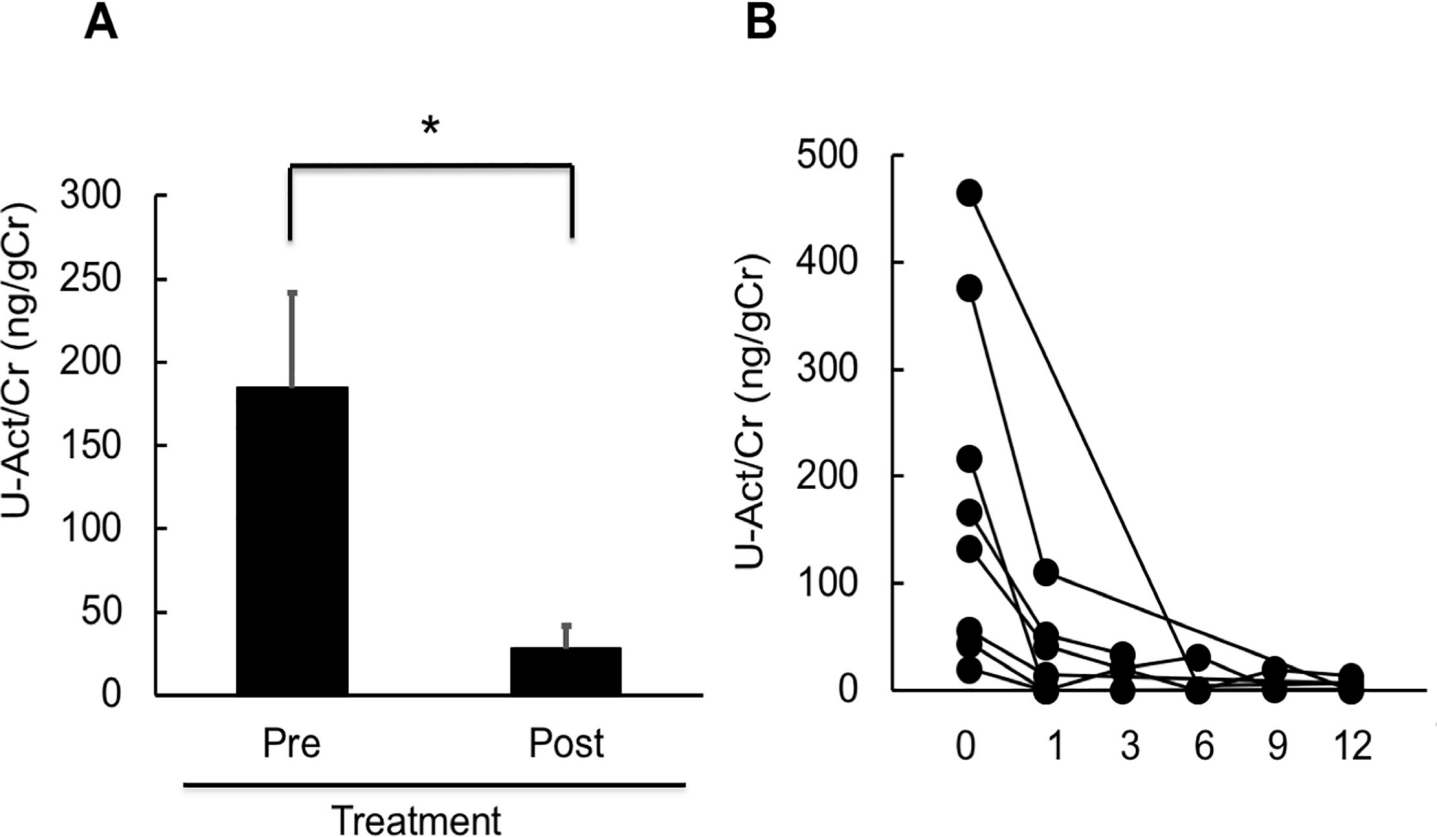
Changes in urinary activin A levels after treatment. A: Urinary activin A levels of patients with first-onset renal AAV before (Pre) and after (Post) treatment (n = 8). *p<0.05. B: Changes in urinary activin A levels in each patient with renal AAV, as measured before and after treatment.

Next, to examine treatment-associated changes in urinary activin A in patients with active AAV, we analyzed urinary activin A levels at 1, 3, 6, and 12 months after treatment in patients with first-onset renal AAV. Urinary activin A concentrations were significantly decreased after treatment (Fig 2A). In 5 of 8 cases, urinary activin A levels fell to a normal range (< 10 ng/gCr) at 1 month after treatment and remained at low levels thereafter (Fig 2B).

When the difference between pre-treatment and 1 month after treatment was analyzed, the rate of change of urinary activin A levels was greater than those of urinary levels of protein, ANCA titer, serum creatinine, and eGFR.

### Correlation of urinary activin A levels with clinical parameters

**Fig 3:**
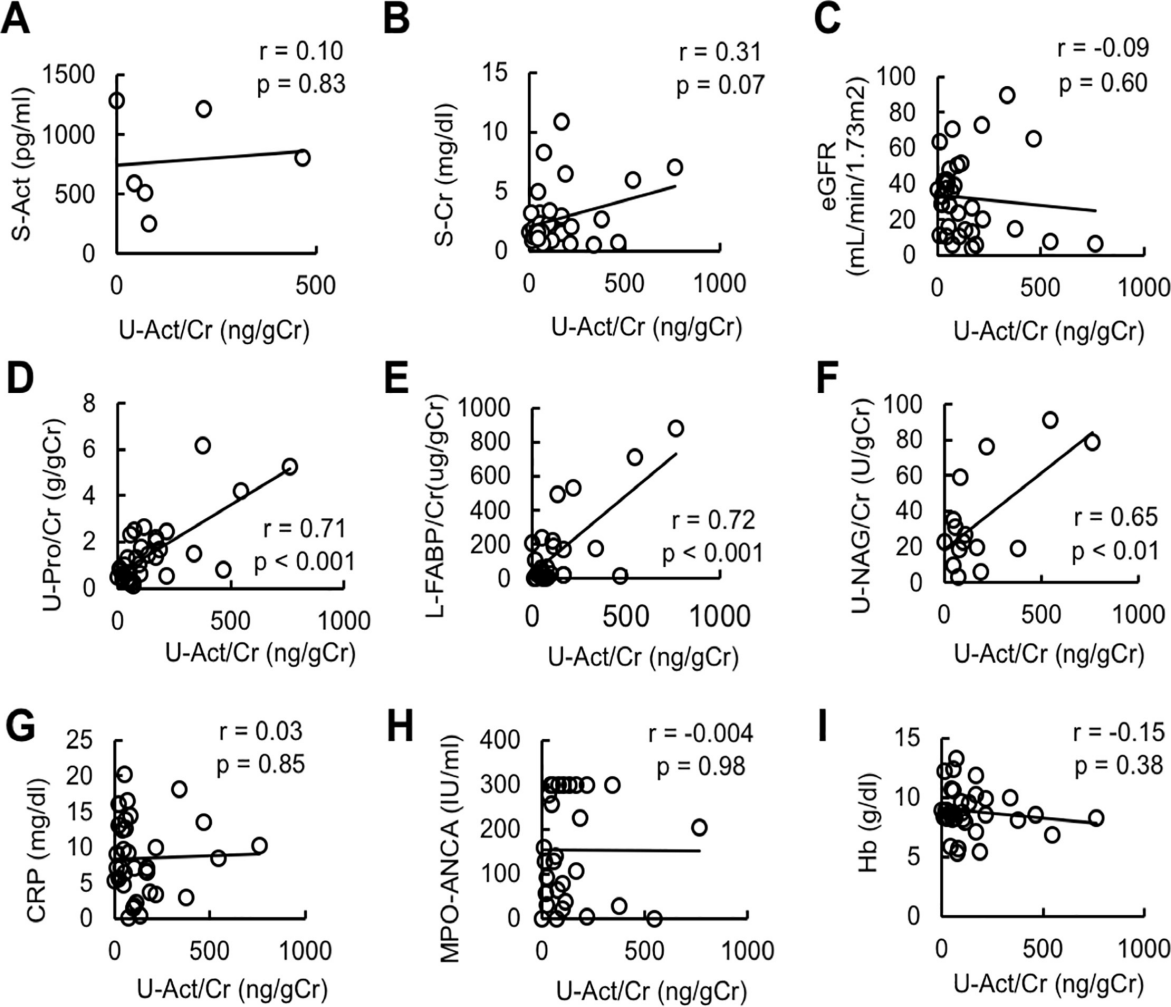
Correlation of urinary activin A level with clinical parameters. A-I: Correlation between urinary activin A and serum activin A (A), serum creatinine (B), eGFR (C), CRP (D), hemoglobin (E), MPO-ANCA (F), urinary protein level (G), urinary NAG (H), and urinary L-FABP (I).

We then analyzed the correlation of urinary activin A levels with clinical parameters. Notably, there was no significant correlation of urinary levels of activin A with serum levels of activin A (831.1±192.8)(ng/gCr), creatinine, eGFR, CRP, and hemoglobin, or with serum ANCA titer (Fig 3A–3F). On the other hand, there was a significant correlation of urinary levels of activin A with urinary levels of protein, NAG, and L-FABP (Fig 3G–3I).

### Correlation of urinary activin A with histological changes in kidney biopsy specimens

**Fig 4:**
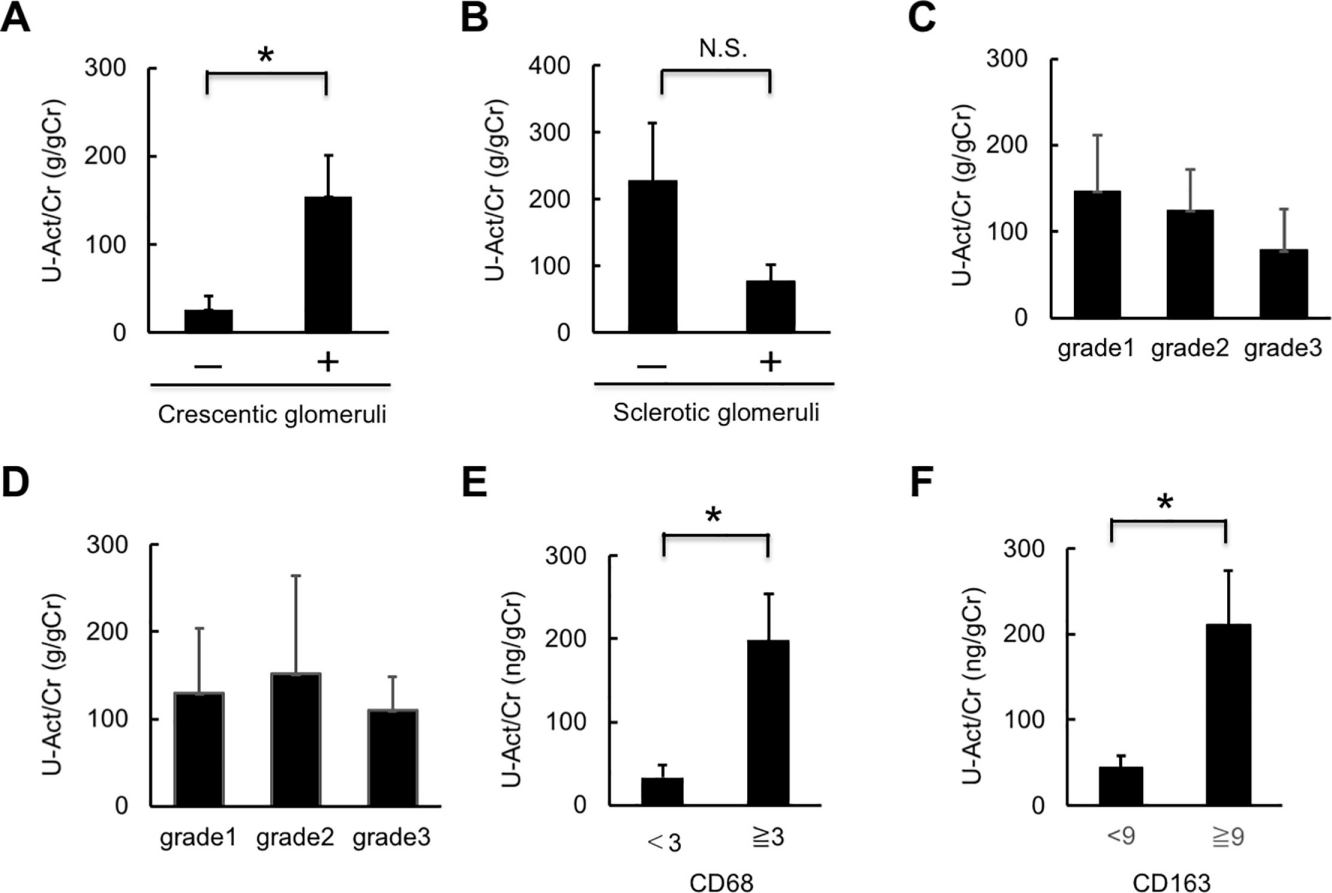
Correlation of urinary activin A level with histological changes in kidney biopsy specimens. A: Urinary activin A levels in patients with renal AAV with (+; n = 11) and without (-; n = 3) cellular crescentic glomeruli. *p<0.05. B: Urinary activin A levels in patients with renal AAV with (n = 9) and without (n = 5) sclerotic glomeruli. N.S., not significant. C, D: Urinary activin A levels in patients with renal AAV with interstitial injuries (grade 1–3) (C) and interstitial cell infiltration (grade 1–3) (D). E: Urinary activin A levels in patients with renal AAV with more than (n = 8) and less than (n = 6) three CD68-positive cells per selected field. *p<0.05. F: Urinary activin A levels in patients with renal AAV with more than (n = 7) and less than (n = 7) nine CD163-positive cells per selected field. *p<0.05.

We next examined the correlation between urinary activin A levels and renal histological changes in patients with renal AAV. Urinary activin A levels were higher in patients with cellular crescentic glomeruli compared to those lacking this damage (Fig 4A), while there was no significant correlation between urinary activin A levels and the presence of sclerotic glomeruli (Fig 4B). There was no significant correlation between urinary activin A levels and the degree of interstitial fibrosis or interstitial cell infiltration (Fig 4C and 4D).

We also analyzed the correlation between urinary activin A level and the degree of CD68- or CD163-positive macrophage infiltration. Most CD68- or CD163-positive macrophages were detected in the interstitium of the kidneys in patients with renal AAV. When the data was divided into two group using median values, urinary activin A levels were associated with the degree of CD68-positive or CD163-positive macrophage infiltration (Fig 4E and 4F).

### Localization of activin A mRNA and protein in the kidneys of the patients with AAV

**Fig 5:**
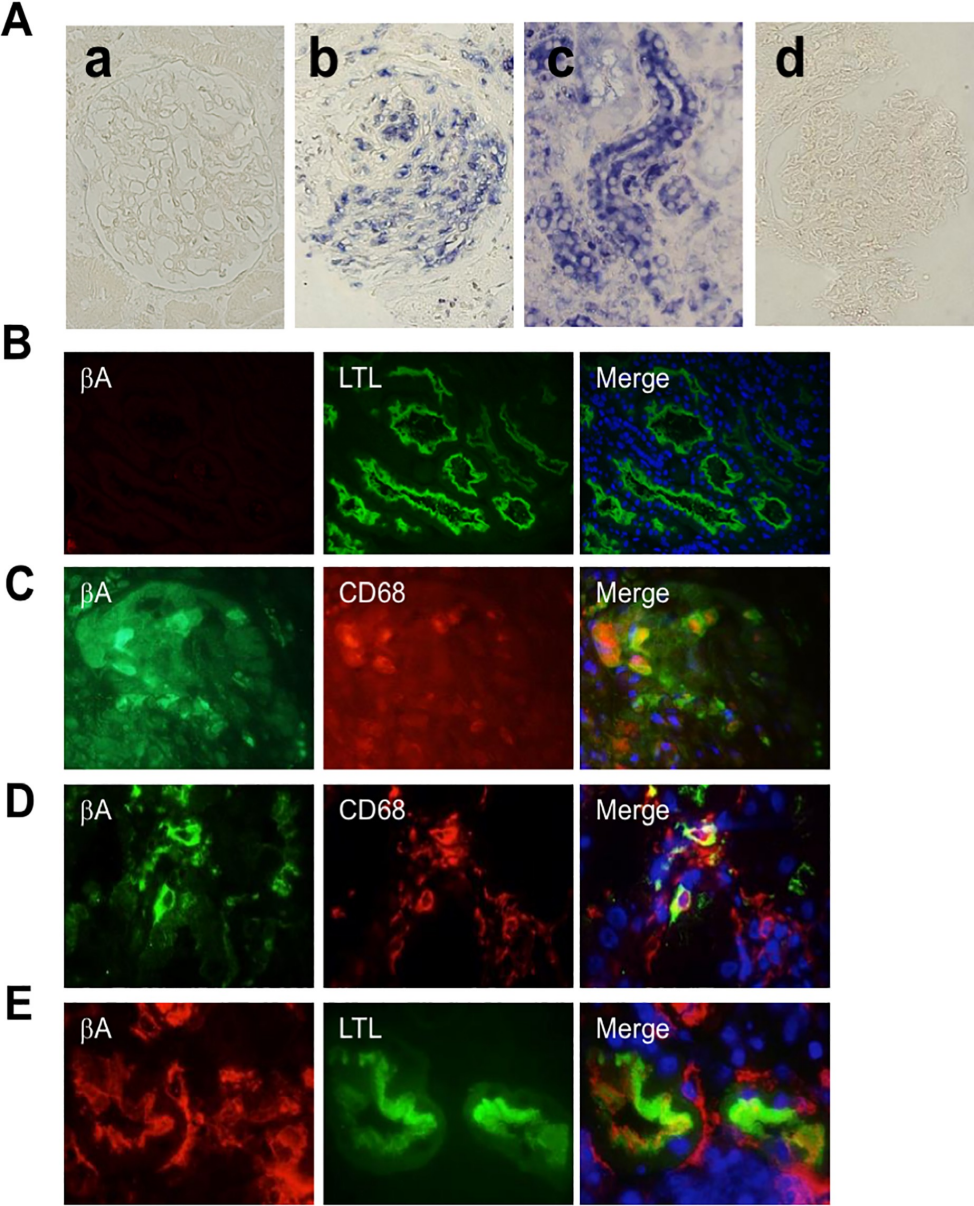
Localization of βA subunit-encoding mRNA and protein in the kidneys of patients with renal AAV. A: Localization of mRNA encoding the βA subunit in kidney specimens of patients with renal AAV was examined by in situ hybridization. Hybridization signals are shown as blue color. Magnification, ×1000. (a) normal glomeruli, (b, d) crescentic glomeruli, (c) tubules. Detection using (a-c) anti-sense probe or (d) sense probe (control). B: Colocalization of activin A βA subunit and LTL in normal kidney specimens obtained from patients who underwent nephrectomy because of renal cancer. βA subunit (red), LTL (green) and DAPI (blue). Magnification, ×400. C, D: Colocalization of βA subunit for activin A and CD68 in glomeruli (B) and interstitium (C) of kidney specimens of patients with renal AAV. βA subunit (green), CD68 (red), DAPI (blue). Magnification, ×400. E: Colocalization of activin A βA subunit and LTL in kidney specimens of renal AAV patients. βA subunit (red), LTL (green) and DAPI (blue). Magnification, ×1000.

To examine the localization of the mRNA encoding the βA subunit of activin A in the kidneys of the patients with AAV, we performed in situ hybridization. Normal kidney specimens obtained from patients who underwent nephrectomy because of renal cancer were used as controls. Hybridization signals were not observed in normal kidneys (Fig 5A-a). In contrast, strong hybridization signals for the βA subunit-encoding mRNA were observed in the kidneys of patients with AAV, primarily in tubular cells and crescent glomeruli (Fig 5A-b and 5A-c). A control experiment using a sense probe showed no hybridization signal in these same specimens (Fig 5A-d).

We also used immunostaining to localize activin A protein expression in renal biopsy specimens obtained from patients with renal AAV. Notably, activin A protein was not detected in normal kidneys (Fig 5B). In contrast, activin A was detected in cellular crescentic glomeruli (Fig 5C), and in infiltrating macrophages in the interstitium (Fig 5D) and apical site of proximal tubules (Fig 5E) in the kidneys of patients with renal AAV.

## Discussion

In this study, we found that urinary activin A concentrations were significantly elevated in patients with renal AAV; this parameter decreased rapidly following immunosuppressive treatment. Urinary activin A was correlated with the levels of urinary tubular injury markers and was significantly higher in patients with renal AAV whose specimens exhibited cellular crescentic glomeruli and extensive macrophage infiltration. We first hypothesize that urinary activin A is derived from glomerular filtered activin A. The molecular weight of activin A is 25 kDa, a size that can be theoretically filtered by glomeruli. However, this idea seems to be less likely, because there was no significant correlation of urinary activin A levels with serum activin A levels (Fig 3A). In situ hybridization demonstrated that βA subunit mRNA for activin A was expressed in proximal tubules and crescentic glomeruli in the kidneys of patients with renal AAV. Activin A was also present in the infiltrating macrophages, suggesting that urinary activin A was derived from the inflamed kidney but not from the blood. Taken together, these results suggest that urinary activin A concentration may be a useful biomarker for monitoring renal inflammation and tubular damage in patients with AAV.

Previous research has identified several urinary biomarkers that may be useful for monitoring AAV activity, including the levels of alpha-1 acid glycoprotein [27], monocyte chemoattractant protein-1 (MCP-1) [27, 28], high mobility group box-1 (HMGB1) [29], and tissue inhibitor of metalloproteinases‐1 (TIMP-1), as well as Matrix metalloproteinase-2 (MMP-2) activity [30]. Recently, the urinary concentration of soluble CD163, a marker of M2 macrophages, was found to be a biomarker for AAV [31]. A combination of urinary soluble CD163 and urinary MCP-1 measurements was reported to be advantageous for diagnosing subtle flares of renal vasculitis [32]. Furthermore, measurement of urinary soluble CD25 and serum soluble CD25 was shown to complement urinary soluble CD163 in the detection of active renal vasculitis [33]. These data suggest that urinary biomarkers reflecting inflammatory cell infiltration are suitable for assessing disease activity in renal AAV. Consistent with this idea, we found that activin A levels were elevated in the urine from patients with active renal AAV; we infer that increased activin A was produced by infiltrating macrophages in the kidneys of these patients. Because activin A in urine is stable for at least five years at -20°C (Takei et al. unpublished observation), we expect that retrospective measurement of urinary activin A in stored samples may be advantageous in the clinical setting.

Expression of kidney injury molecule-1 (KIM-1), a type-1 transmembrane protein with an immunoglobulin and mucin domain, is significantly increased in the proximal tubule after acute kidney injury [34]. Therefore, urinary KIM-1 has been proposed as a biomarker for renal proximal tubule injury [34]. Recently, KIM-1 also was found in the urine of patients with active renal AAV [27], suggesting that tubular injury markers will be useful for detecting the presence of kidney injury in patients with AAV. Recently, we reported that urinary activin A acts as a useful biomarker for acute kidney injury [17]. Given that the expression (at both the mRNA and protein level) of activin A was elevated in the renal tubules of the kidneys of patients with renal AAV (Fig 5), urinary activin A is expected to reflect both renal inflammation and tubular injury in patients with renal AAV.

The present study demonstrated that urinary activin A was useful for distinguishing patients with renal AAV from those with non-renal AAV. It is likely that urinary activin A levels reflect the severity of renal AAV, although it remains unclear whether urinary activin A would be of use as a renal prognosis marker or as a signal for renal relapse. Combination measurement of urinary activin A with other urinary biomarkers such as soluble CD163, soluble CD24, and MCP1 is expected to facilitate clinical monitoring of the status of patients with renal AAV. Limitation of this study is that it remains unknown if infection of urinary tract affects urinary activin A levels. Urinary activin A was also detectable not only in patients with renal AAV, but also in patients with non-inflammatory kidney diseases (S1 Fig). Further study will be needed to clarify the pathophysiological role of activin A in the development of various kidney diseases.

## Supporting information

S1 Fig Urinary activin A levels in patients with various kidney diseases. Urinary activin A levels in healthy controls (HC) (n = 8), IgA nephropathy (IgAN) (n = 81), lupus nephritis (LN) (n = 80), hypertensive nephrosclerosis (HN) (n = 31), minimal change nephrotic syndrome (MCNS) (n = 21), DM nephropathy (DM-N) (n = 20), tubulointerstitial nephritis (TIN) (n = 9), membranoproliferative glomerulonephritis (MPGN) (n = 8), polycystic kidney disease (PCK) (n = 8), and Alport syndrome (Alport) (n = 4).
(TIFF)

S1 Methods Patients. This study enrolled the patients with various kidney diseases including IgA nephropathy (IgAN) (n = 81), lupus nephritis (LN) (n = 80), hypertensive nephrosclerosis (HN) (n = 31), minimal change nephrotic syndrome (MCNS) (n = 21), DM nephropathy (DM-N) (n = 20), tubulointerstitial nephritis (TIN) (n = 9), membranoproliferative glomerulonephritis (MPGN) (n = 8), polycystic kidney disease (PCK) (n = 8), and Alport syndrome (Alport) (n = 4) who were treated in Gunma University Hospital from November 2011 to March 2018. Urinary activin A concentration was quantified by enzyme-linked immunosorbent assay (ELISA) according to the manufacturer’s instructions (Kit No. DAC00B; R&D Systems Inc., Minneapolis, MN). This study was approved by the ethical committee on human research of Gunma University Graduate School of Medicine (Approval numbers 855 and 15–104). Written informed consent was obtained from all patients.
(DOCX)
