# Supplementary figures and images for: Urinary Activin A is a novel biomarker reflecting renal inflammation and tubular damage in ANCA-associated vasculitis
Source: PLoS One. 2019 Oct 15;14(10):e0223703. doi: 10.1371/journal.pone.0223703 (PMC6793943; doi:10.1371/journal.pone.0223703)

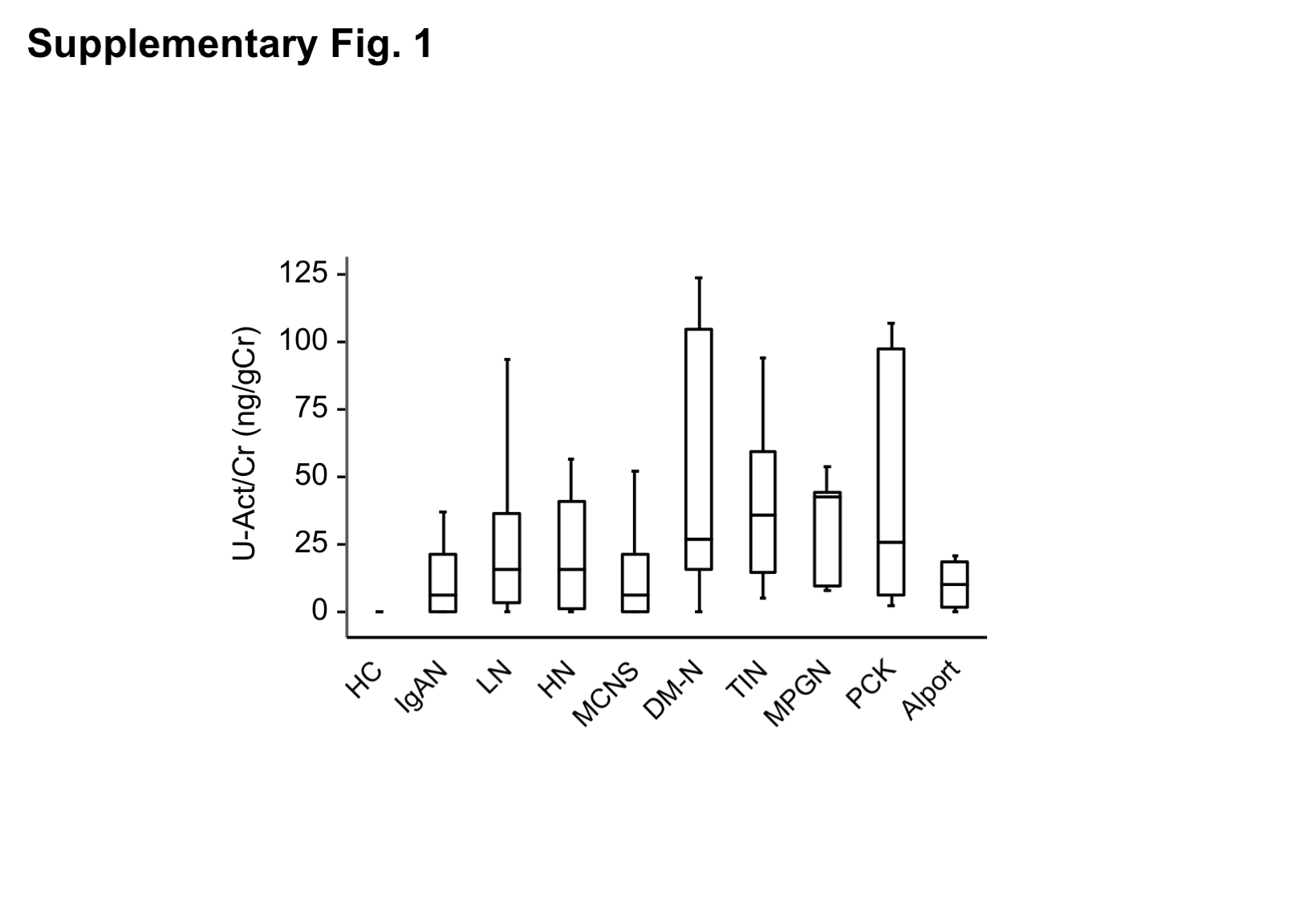

Supplement: S1 Fig — Urinary activin A levels in healthy controls (HC) (n = 8), IgA nephropathy (IgAN) (n = 81), lupus nephritis (LN) (n = 80), hypertensive nephrosclerosis (HN) (n = 31), minimal change nephrotic syndrome (MCNS) (n = 21), DM nephropathy (DM-N) (n = 20), tubulointerstitial nephritis (TIN) (n = 9), membranoproliferative glomerulonephritis (MPGN) (n = 8), polycystic kidney disease (PCK) (n = 8), and Alport syndrome (Alport) (n = 4). (TIFF) [file pone.0223703.s001.tiff]
