# Supplementary material for: Urinary Activin A is a novel biomarker reflecting renal inflammation and tubular damage in ANCA-associated vasculitis
Source: PLoS One. 2019 Oct 15;14(10):e0223703. doi: 10.1371/journal.pone.0223703 (PMC6793943; doi:10.1371/journal.pone.0223703)
Supplement: S1 Methods — This study enrolled the patients with various kidney diseases including IgA nephropathy (IgAN) (n = 81), lupus nephritis (LN) (n = 80), hypertensive nephrosclerosis (HN) (n = 31), minimal change nephrotic syndrome (MCNS) (n = 21), DM nephropathy (DM-N) (n = 20), tubulointerstitial nephritis (TIN) (n = 9), membranoproliferative glomerulonephritis (MPGN) (n = 8), polycystic kidney disease (PCK) (n = 8), and Alport syndrome (Alport) (n = 4) who were treated in Gunma University Hospital from November 2011 to March 2018. Urinary activin A concentration was quantified by enzyme-linked immunosorbent assay (ELISA) according to the manufacturer’s instructions (Kit No. DAC00B; R&D Systems Inc., Minneapolis, MN). This study was approved by the ethical committee on human research of Gunma University Graduate School of Medicine (Approval numbers 855 and 15–104). Written informed consent was obtained from all patients. (DOCX) [file pone.0223703.s002.docx]

**Supplementary Methods**

**Patients**

This study enrolled the patients with various kidney diseases including IgA nephropathy (IgAN) (n=81), lupus nephritis (LN) (n=80), hypertensive nephrosclerosis (HN) (n=31), minimal change nephrotic syndrome (MCNS) (n=21), DM nephropathy (DM-N) (n=20), tubulointerstitial nephritis (TIN) (n=9), membranoproliferative glomerulonephritis (MPGN) (n=8), polycystic kidney disease (PCK) (n=8), and Alport syndrome (Alport) (n=4) who were treated in Gunma University Hospital from November 2011 to March 2018. This study was approved by the ethical committee on human research of Gunma University Graduate School of Medicine (Approval numbers 855 and 15-104). Written informed consent was obtained from all patients.
